# Challenges and Tendencies of Automatic Milking Systems (AMS): A 20-Years Systematic Review of Literature and Patents

**DOI:** 10.3390/ani11020356

**Published:** 2021-01-31

**Authors:** Alessia Cogato, Marta Brščić, Hao Guo, Francesco Marinello, Andrea Pezzuolo

**Affiliations:** 1 Department of Land, Environmental, Agriculture and Forestry, University of Padova, 35020 Legnaro (PD), Italy; alessia.cogato.1@phd.unipd.it (A.C.); francesco.marinello@unipd.it (F.M.); 2 Department of Animal Medicine, Production and Health, University of Padova, 35020 Legnaro (PD), Italy; marta.brscic@unipd.it; 3 College of Land Science and Technology, China Agricultural University, Beijing 100083, China; guohaolys@cau.edu.cn

**Keywords:** automation, dairy farming, milking, robotic milking, precision livestock farming

## Abstract

**Simple Summary:**

Automatic milking systems (AMS) are spreading rapidly among farms. The contribution of AMS to speeding up the milking process and increasing yield is unquestionable. Nonetheless, thanks to continuous research, AMS have shown the potential to improve animal welfare. In this review, we carried out a comprehensive systematic review of the scientific and industrial research on AMS over the last 20 years. The objectives of this study were to identify the tendencies and gaps of research on AMS and to help the scientists addressing future research. The results showed that, despite the interest in milk production, some gaps remain on the improvement of milk quality. Moreover, future research tendencies will likely be related to animal welfare, sensing technologies and the Internet of Things (IoT) systems.

**Abstract:**

Over the last two decades, the dairy industry has adopted the use of Automatic Milking Systems (AMS). AMS have the potential to increase the effectiveness of the milking process and sustain animal welfare. This study assessed the state of the art of research activities on AMS through a systematic review of scientific and industrial research. The papers and patents of the last 20 years (2000–2019) were analysed to assess the research tendencies. The words appearing in title, abstract and keywords of a total of 802 documents were processed with the text mining tool. Four clusters were identified (Components, Technology, Process and Animal). For each cluster, the words frequency analysis enabled us to identify the research tendencies and gaps. The results showed that focuses of the scientific and industrial research areas complementary, with scientific papers mainly dealing with topics related to animal and process, and patents giving priority to technology and components. Both scientific and industrial research converged on some crucial objectives, such as animal welfare, process sustainability and technological development. Despite the increasing interest in animal welfare, this review highlighted that further progress is needed to meet the consumers’ demand. Moreover, milk yield is still regarded as more valuable compared to milk quality. Therefore, additional effort is necessary on the latter. At the process level, some gaps have been found related to cleaning operations, necessary to improve milk quality and animal health. The use of farm data and their incorporation on herd decision support systems (DSS) appeared optimal. The results presented in this review may be used as an overall assessment useful to address future research.

## 1. Introduction

In the last years, the dairy industry is experiencing a constant increase in herd sizes and a concurrent declining workforce. To face these changes, farmers are increasingly adopting automation and precision livestock farming technologies [1,2,3]. 

Automation in dairy farming is developed for several monitoring and control applications, such as herd-management, milk production, feed distribution, environmental control and animals’ health/behaviour assessment [4,5,6]. The more advanced use of automation involves robotic systems or intelligent machines capable of interacting with their work environment without direct human control [7]. 

In this last scenario, the introduction of Automatic Milking Systems (AMS) was one of the most significant technological developments in the dairy sector [8,9]. AMS can be considered not only as an alternative to traditional milking systems but also as a new and general approach to manage dairy herd health and production efficiency [10].

The first commercial AMS in dairy farms were introduced in the Netherlands in the early 1990s [10], and by 2020, the AMS manufacturers estimated a worldwide adoption of 50,000 units [6], mainly concentrated in Europe (90%), Canada (9%) and other countries (1%) [11]. In particular, in Europe, the adoption of AMS is more marked, and it is expected that by 2025, 50% of dairy cows in North-Western Europe will be equipped with AMS [12].

Along with the increasing adoption of AMS technology, a large number of research works have reported analyses of its consequence on specific aspects, such as milk yield/quality [9,10,11,13], animal behaviour/health/welfare [14,15,16], herd management [17,18], performance and labour efficiency [19,20]. Some studies reported an increase in milk production of 2 to 12% in cows milked 2+ times per day in AMS compared with cows milked twice per day in traditional milking parlours [10,21]. However, other studies did not show an increase in milk production, especially for primiparous cows [10,22]. 

Regarding the AMS performance, Calcante et al. [23] showed that the electric consumption is mainly conditioned by farm management rather than machine characteristics/architectures. However, compared with the first AMS, in recent years the performance has been improved. Salfer et al. [20] disserted that milk yield and labour savings are the two most important factors to define whether AMS units are more profitable than traditional milking parlours. Thanks to AMS technology, livestock farmers are freed from the milking process and its rigid schedule and focus on the supervision of herd and farms [24].

Compared to the past generation AMS, the implementation of sensors and technologies is critical not only for the efficiency of recent AMS but also for the “animal–AMS” relationship [25]. Robotic technology and sensors, especially those monitoring udder health, milk quality, reproductive status, feed intake and body weight changes, provide exhaustive information about each animal at each milking process [26,27]. Consequently, health and production status of every animal can be characterized in greater depth. However, the development of high-tech AMS makes decision support systems (DSS) necessary to help livestock farmers in decision-making and assist in the early detection of animal diseases or abnormal milk production.

Although several studies were carried out on specific and general aspects related to AMS, a long-term review of both industrial and scientific research on AMS is missing to the best of our knowledge. This review aims to provide a systematic and critical evaluation of AMS’s published documentation, starting from their introduction in the commercial dairy barns. The analysis was carried out both from the academic and the industrial perspective. Such study may provide a useful tool to examine the state of the art of AMS implementation and serve as a method to observe tendencies or areas needing further research for the improvement of the dairy sector, particularly for dairy farmers.

This paper presents a systematic review of 20-years of industrial and scientific research (2000–2019) in the AMS sector, examining tendencies and gaps and identifying possible future developments. 

## 2. Materials and Methods

The analysis of the state of the art of the research on AMS was conducted through a systematic review. The literature of the last 20-years (2000–2019) was considered. The approach applied for this review has been already used by several authors. The aim was to map the tendencies and gaps of the research topic from a theoretical and methodological point of view [28]. Unlike the majority of the systematic reviews, this study analysed papers and patents concurrently, thus providing a comprehensive overview of the topic. The approach applied enabled to track the linkage between scientific research and patented technology. Prisma Flow Chart [29] adapted to agricultural research [30] was adopted.

### 2.1. Scoping

The analysis identified papers and patents released in the last two decades dealing with AMS. After the extraction of the documents, the first analysis carried out was an overview of previous reviews. This examination aimed to define the areas that have aroused greater interest so far. A total of 30 documents were identified, limiting the search query to reviews. Two of these documents were not reviews but analysis of massive data collected within experiments [31,32]. Therefore, they were removed from the reviews list and processed as research papers. The reviews were organised in a spreadsheet and examined for year, topic and typology of review (qualitative or quantitative). The spreadsheet is shown in the Results section.

### 2.2. Planning

The papers were extracted from the Scopus database (www.scopus.com), using a custom query in the advanced search tool to limit the field of interest. The patents were searched on the patent server EspaceNet (www.epo.org). The queries used to search papers and patents included combinations of keywords and Boolean operators. Several synonyms commonly adopted to define the AMS were combined. Only “Title, Abstract and Keywords” were considered for the papers search, and “Title and Abstract” for the patents search (Table 1). 

### 2.3. Identification/Search and Screening

Only scientific papers published in English were considered for further analysis. The patents did not need any further implementation of the pre-defined search strategy and once extracted, they were organised in five groups of four years each. The management of the papers and patents extracted was done using the Mendeley tool. Duplicates were removed, and the information of the articles was updated.

### 2.4. Eligibility Criteria

Setting the inclusion and exclusion criteria is essential to exclude the risk of bias. Koutsos et al. [30] proposed a classification of the research papers based on their strength of evidence. According to their categorisation, opinion papers, conference papers and workshops provide low strength of evidence. For this reason, the search was limited to “articles”, “reviews” and “book chapters”.

### 2.5. Text Mining

The words of Title, Abstract and, in the case of papers, Keywords were analysed with the text mining process. Text mining aims to derive meaningful numeric indices describing the research tendencies. The content of Titles, Keywords and Abstract was pre-processed to improve the quality of information retrieval following the protocol established by Kannan and Gurusamy [33]. The first step was to join compound words (i.e., air intake) to preserve their meaning. Then, we applied the tokenisation process, which consists of removing meaningless features, such as punctuation marks, web sites, numbers and symbols. The result of the tokenisation was a list of single words which was perfected deleting the low-frequency words (appearing one or two times) and connectors. Then, the word-sense disambiguation was implemented to clarify the ambiguity of acronyms. Lastly, the stemming process was used to obtain the final list of words. The process consists of including in a single lemma all variant forms of the same word (i.e., images and imaging, or udder and teats). 

### 2.6. Cluster Analysis

After the pre-processing, the words included in the dataset were grouped into four clusters: “Animal”, “Process”, “Technology” and “Components”. Clustering was done based on terms analysis. The words were analysed according to the most frequent usage context. Once we established the main topics of the documents extracted and the words’ technological aspects, we classified each term resulting from text mining into one of the four clusters. The cluster analysis consisted of examining the evolution of the four clusters over the years and comparing the different interest arouse by of papers and patents. Then, a words frequency analysis was carried within each cluster to identify the tendencies, trends and gaps over the observed period. Text mining and clustering were carried out on the complete dataset, i.e., papers (articles, reviews and book chapters) and patents. A further analysis consisted of investigating the geographical distribution of the affiliation of contributors and applicants to determine the top contributing countries. Last, the co-correlation of topics was examined by counting the number of intercorrelations between words contained in Title, Abstract and Keywords. The analyses were carried out using Microsoft Excel, Gephi 0.9.2 (Gephi^®^ Consortium, Compiegne, France) and GraphPad Prism 8.0.0 (GraphPad Software, Inc.; La Jolla, CA, USA) software.

## 3. Results

### 3.1. Scientific Research: Papers Review

**Figure 1:**
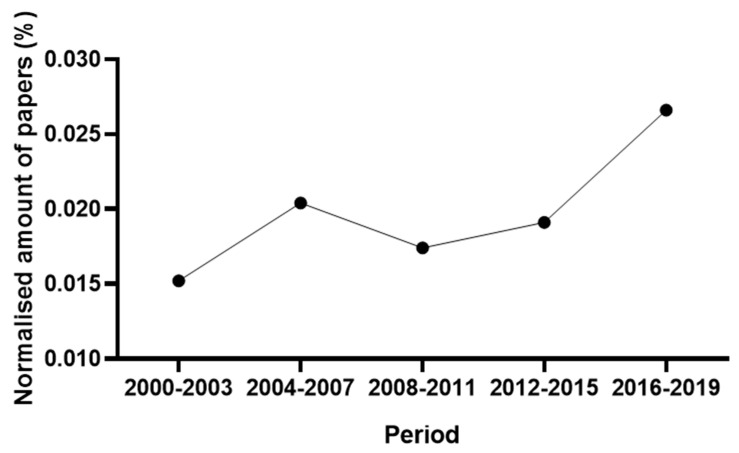
Papers: course of publications over the observed period. The number of papers was normalised on the total amount of documents in subject area “Agricultural and Biological Sciences”.

A total of 585 papers, comprising articles, reviews and book chapters, were identified from 2000 to 2019 using the custom search query. Figure 1 shows the course of the publications over the observed period after normalisation on the total amount of documents in subject area “Agricultural and Biological Sciences”. The normalisation was carried out dividing the number of publications extracted using the search query in the four-year periods by the total amount of publications classified under the aforementioned subject area in the Scopus database. This procedure aimed to avoid confusing the trend of publications on the AMS with the constantly growing trend experienced by research papers in the Scopus database’s agricultural and biological area. The interest of the scientific community in the AMS topic grew very fast from the first to the second four-year period. Then, after a slight contraction, a constantly growing trend was observed. 

#### 3.1.1. Previous Reviews

A total of 28 reviews were yielded from 2000 to 2019: 22 were traditional qualitative reviews, two used a systematic approach, two were questionnaire-based surveys, one was a meta-analysis and one was a quantitative review based on data collected from several institutions. The reviews focused on several aspects: eleven reviews were related to health aspects, six provided a general overview of the AMS topic, five dealt with specific phases of the production process, three were related to ethics, two to sensing technologies and one combined sensors with health issues. For the sake of consistency, the reviews were classified according to the clusters identified in this review (Table 2). 

#### 3.1.2. Papers Tendencies

**Figure 2:**
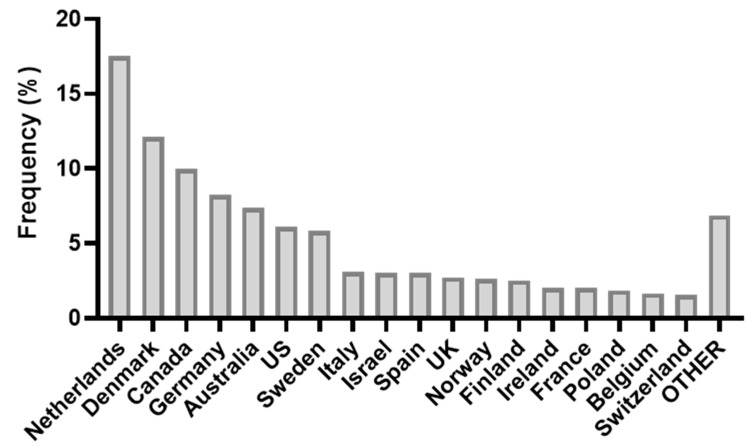
Papers: geographical distribution of authors’ affiliations.

The geographical distribution of the affiliations (Figure 2) showed that the top contributors were the Netherlands, Denmark, Canada, Germany, Australia, US and Sweden (17.5%, 12.1%, 10.0%, 8.2%, 7.4%, 6.1% and 5.8% of the authorships, respectively). All continents were involved in the literature concerning AMS, despite low contribution from Asian and African countries.

**Figure 3:**
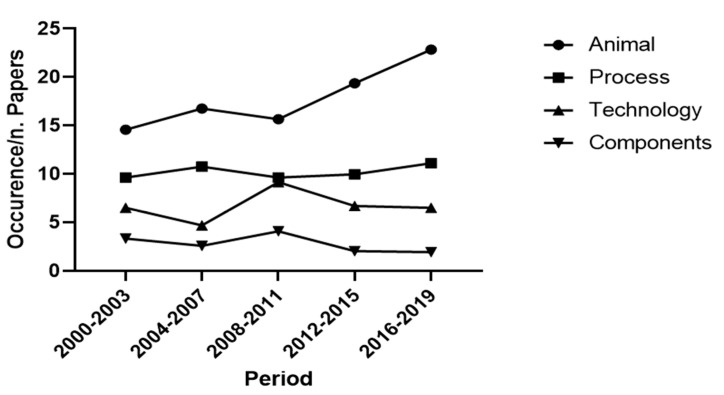
Papers: course of the clusters over the last two decades. The weight of the cluster for each four-year period was calculated as the ratio between the occurrence of words belonging to a cluster and the total amount of papers.

When analysing the complete database (articles, reviews and book chapters), we identified four conceptual clusters were namely “Animal”, “Process“, “Technology“ and “Components“. The cluster “Animal” included words related to animal’s health, behaviour and anatomy. “Process” contained words associated with the milking process and its sustainability. “Technology” consisted of the ensemble of lemmas dealing with the implementation of technologies, sensors and analysis approaches in the farms. Last, “Components” included words describing the technical elements composing the AMS. The analysis of the course of the four clusters over the years showed that the aspects related to the animals raised an increasing interest over the observed period. The trend of the cluster “Process” was more constant, despite a slight increase in the last eight years. The topics related to the technical aspects, including the clusters “Technology” and “Components”, showed a constant trend with a peak in the intermediate period (Figure 3). As shown in Table 3, the largest cluster was “Animal” (49%), followed by “Process” (27%), “Technology” (17%) and “Components” (7%). 

**Figure 4:**
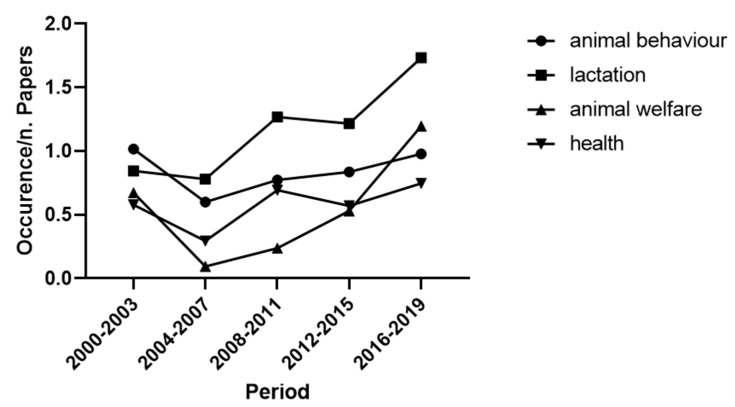
Papers: course of the most representative subjects of cluster “Animal” from 2000 to 2019.

The results from the analysis for cluster “Animal” showed the high relevance of topics related to the animal health and welfare (i.e., mastitis, 4.5%; behaviour 4.1%; welfare 3.2%). However, the highest frequency words were useful for focusing the topic, but the examination of lower frequency words was more effective to describe research tendencies over the last 20 years. For example, the high frequency of the terms “cattle” and “udder” can be ascribable to their frequent inclusion within the keywords of papers on AMS. Besides, as the feed dispenser is an essential component of AMS, the use of the words “feed” and “forage” may be over-general to be interpreted. Therefore, we focused the attention on “lactation”, “animal welfare”, “health” (not shown in Table 3, as its frequency was 2.9%) and “animal behaviour” (Figure 4). The trend of the first three lemmas over the years was positive, while the trend of “animal behaviour” was negative (-4% from 2000 to 2019). However, despite a sharp decrease in the second four-year period, the topic has been growing steadily until 2019.

**Figure 5:**
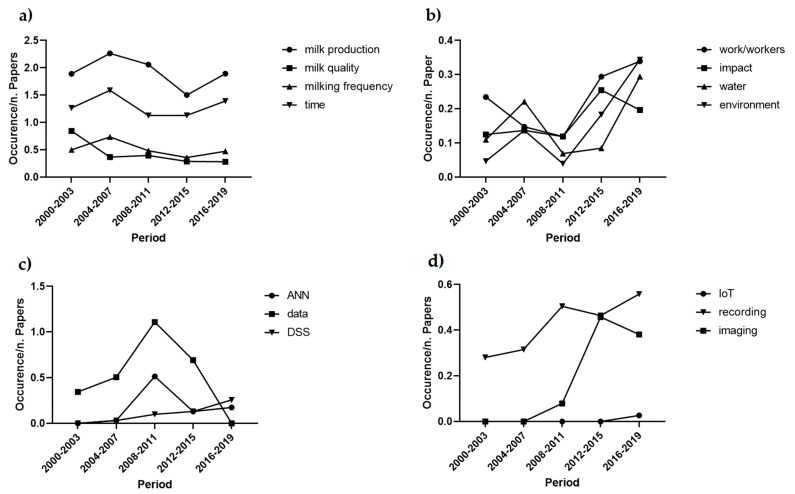
Papers: course of the most representative subjects of clusters “Process” and “Technology” from 2000 to 2019. (**a**) tendencies of the production process; (**b**) tendencies of the production sustainability; (**c**) the shift from data to models and decision support (ANN = Artificial Neural Networks; DSS = Decision Support Systems); (**d**) tendencies of new technologies (IoT = Internet of Things).

The highest frequency words of cluster “Process” were associated to the production process (i.e., milk production, 18.5%; time, 12.6%; milk flow, 4.8%; milk frequency, 4.7%). An in-depth analysis of the different aspects of the milking process allowed to define the main objectives of the studies on AMS. During the observed period, the milk yield was the priority over the other production factors. The milking time followed a rather similar course as milk yield. The focus on milk quality and milking frequency was rather stable over the last two decades (Figure 5a). Although not appearing within the cluster’s highest frequency words, some lemmas showed a clear positive trend of the concern on sustainability (Figure 5b). These lower frequencies lemmas revealed a growing interest in sustainable processes, such as workers, environment, impact and water. Due to their lower frequencies (2.5%, 1.9%, 1.8% and 1.7%, respectively), these topics were not reported in Table 3.

The analysis of cluster “Technology” showed that the highest frequency subjects in this area were related to data analysis and modelling (i.e., data, 12.7%, analysis, 11.4% and model 10.6%). The detailed investigation of the tendencies over the observed period revealed a gradual shift of scientific literature interest from data to DSS. Although emerging topics related to the technological development, such as Artificial Neural Networks (ANN) and DSS, exhibited low frequencies (2.7% and 1%, respectively), we report their growing trend in Figure 5c. Moreover, the analysis showed the increasing relevance of new technological implements, such as imaging and recording techniques (3.3% and 6.9%, respectively), and the growing interest towards the internet of things (0.2%) (Figure 5d).

The highest frequency words of the cluster “Components” were cell count, voluntary milking system and parlour (40.6%, 26.6% and 14.0%, respectively). No notable trends were detected within the words of this cluster, as they remained relatively stable over the observed period.

**Figure 6:**
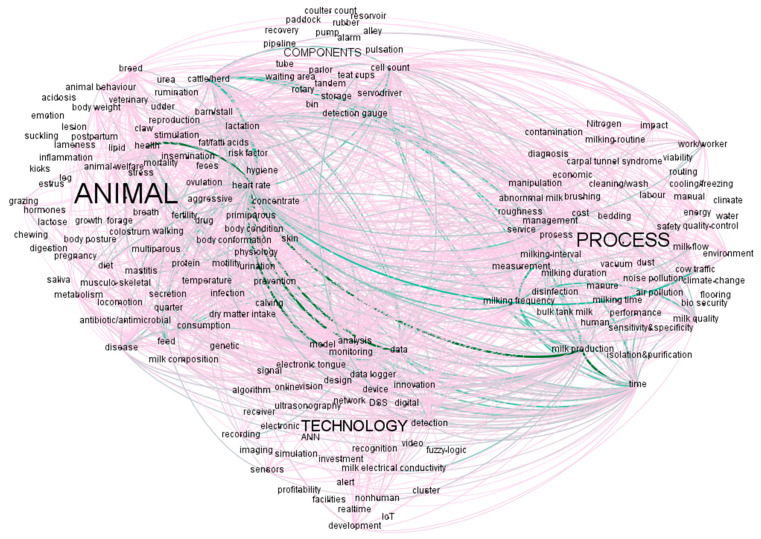
Papers: overview of the words grouped into clusters. The size of the label of the cluster name is proportional to the cluster weight. Co-occurrence of topics is represented by edges, which thickness and darker color of the lines indicate a larger number of connections. Co-occurences lower than 4 were not represented.

The results of the analysis of the co-correlation of topics (Figure 6) enabled us to identify some critical interconnections. Specifically, the couples of topics inter-associated more frequently were health and heart rate; analysis and heart rate; model and heart rate; data and heart rate; milk production and heart rate; time and milk production.

### 3.2. Industrial Research: Patents Tendencies

**Figure 7:**
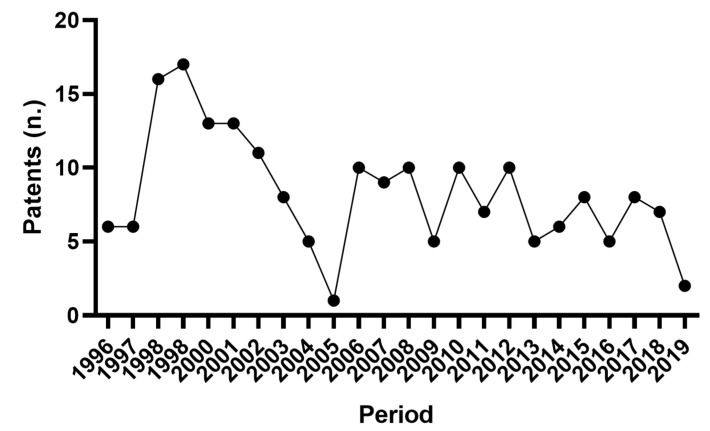
Patents: course of patents released over the observed period.

A total of 217 patents were released from 2000 to 2019. Figure 7 shows the patents yielded over the observed period. The graph was built starting from 1996, as the employment of AMS in the farms dates to the ‘90s. This procedure helped us to better understand the interest of the industrial sector in the AMS technologies. The highest number of patents was released in 1998–1999. Then, after a sharp decrease, the patent registration was stabilised starting from 2006.

**Figure 8:**
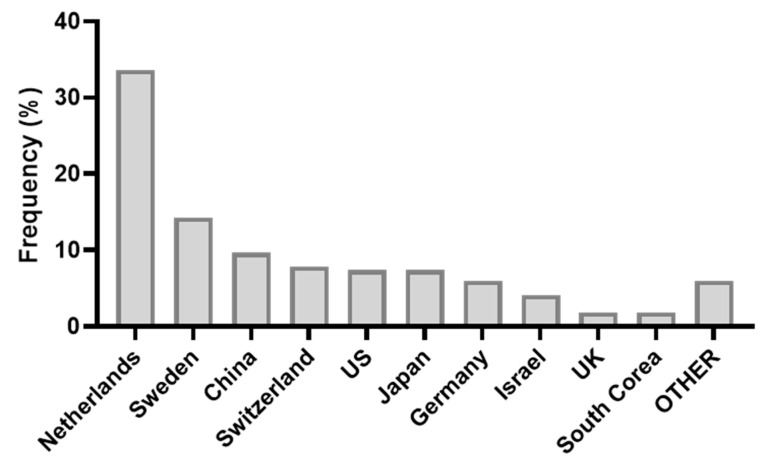
Patents: geographical distribution of applicants’ affiliations.

The analysis of the geographical distribution of the applicants (Figure 8) showed that the highest number of patents was granted by the Netherlands (33.6%), followed by Sweden (14.3%) and China (9.7%).

**Figure 9:**
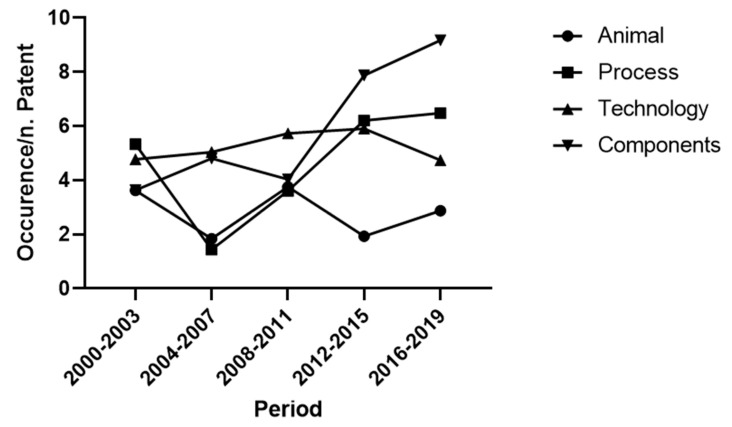
Patents: course of the clusters over the last two decades. The weight of the cluster for each four-year period was calculated as the ratio between the occurrence of words belonging to a cluster and the total amount of patents.

The same conceptual clusters identified in the analysis of papers were considered. The analysis of the tendency of the four clusters over the years showed that in the first period the industrial research focused on the technological development of the AMS. Afterwards, the focus shifted towards the improvement of the process efficiency and the technological updating of the components. Despite the lower frequency of cluster “Animal”, the interest on this topic was constant over the observed period (Figure 9).

As shown in Table 4, the largest cluster was “Components” (30%), followed by “Technology” (29%), “Process” (25%) and “Animal” (16%). 

**Figure 10:**
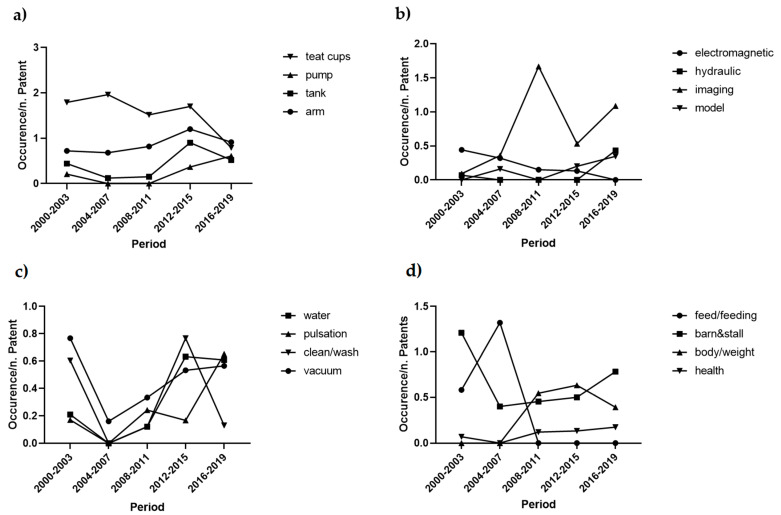
Patents: course of the most representative topics of (**a**) cluster “Components”; (**b**) cluster “Technology”; (**c**) cluster “Process” and (**d**) cluster “Animal” from 2000 to 2019.

Regarding the single clusters, the frequency analysis of cluster “Components” focused on the highest frequency meaningful words: teat cups, milking arm, tank and pump (29.0%, 14.0%, 7.1% and 3.6%, respectively). The analysis showed that, after the initial implementation of teat-cups, the industry spent the second decade of the observed period improving tanks and pumps. Moreover, the number of patents focusing on the milking arm increased constantly until 2015 and the showed a slight decrease (Figure 10a). 

Given the general meaning of some of the highest frequency words, the in-depth analysis of the cluster “Technology” focused on some topics not appearing in Table 4. The topics analysed were imaging, electromagnetic, hydraulic control and models (12.3%, 4.1%, 1.5% and 2.0%, respectively). The topic with the highest increase in the last decade within the cluster “Technology” was imaging techniques. Moreover, the interest in models showed constant growth from 2000 to 2019. As regards the movement, the results showed a slight difference between electromagnetic and hydraulic control (Figure 10b). The cluster “Process” showed some recurrent words. We focused our attention on the increasing relevance of cleaning operations analysing the course of the words water and cleaning/washing (6.3% and 5.0%, respectively). In the face of an increase in the frequency of patents dealing with water management, the words “cleaning” and “washing” showed a fluctuating trend. Then, the milking process was analysed, by examining the course of vacuum and pulsation technologies (10.0% and 4.8%, respectively) which aroused growing interest over the observed the 20-year observed period, mainly from 2004. The course of the topics of cluster “Process” are shown in Figure 10c.

The smallest cluster was the one related to “Animal”, which comprised few words. For this reason, some of them were examined jointly, as they referred to linked topics (Figure 10d). The highest frequency words analysed to describe the tendencies of the patents were barn/stall (21.7%), feed/feeding (11.4%), body/weight (9.1%) and health (2.2%). The analysis showed that the initial focus on the technical aspects (barn/stall and feed/feeding) was subsequently moved towards the animal welfare (body condition/weight and health). However, the stall has continued to be an essential research topic.

## 4. Discussion

This study analysed a total of 802 documents (papers and patents) to provide a comprehensive view of the state of the art, gaps and tendencies of the research on the AMS. The number of published papers showed a spike in 2004–2007 (Figure 1) attributable to the full implementation of AMS in farms during those years. The farms worldwide using AMS increased from 800 to 8000 in the period of 2000–2007 [58]. The contributors were mainly from countries where dairy farms are more intensely managed (Figure 2). It is worth noting that the long tradition and experience of the Netherlands in livestock research is likely the reason for the large number of papers yielded from this country. In fact, the first commercial AMS were installed in this country. 

The in-depth examination of previous reviews showed that almost half of the reviews published over the last two decades were dedicated to animal health. Although previous general reviews on the AMS have been published, the analysis showed that a comprehensive study considering both papers and patents with a quantitative approach was still missing (Table 2). 

Observing the cluster tendencies over the last 20 years (Figure 3) it can be deduced that the main focuses of research were related to the animal aspects and the implementation of the process. As regards the animal cluster, several aspects were considered, thanks to the high frequencies of terms related to health (udder and mastitis, 7.8% and 4.5%, respectively), feeding (feed and forage, 7.6% and 4.4%, respectively) and welfare/behaviour, 3.2% and 4.1%, respectively (Table 3). Monitoring lactation is critical as it allows the control of milk yield dynamics and AMS performance. Several models simulating lactation curves have been tested in conventional milking systems, however, prediction models for AMS are still being developed [13]. In this case, models could assist in monitoring the lactation curve and minimising cows’ responses to physiological and environmental stress [59].

The growing interest in the animal topic also involved many factors related to animal health, welfare and behaviour (Figure 4). Recent studies highlighted that animal welfare is becoming the focus of societal and political attention [60,61]. Although animal welfare has not been defined unequivocally, according to the World Organisation for Animal Health (OIE) “Animal welfare means how an animal is coping with the conditions in which it lives. An animal is in a good state of welfare if (as indicated by scientific evidence) it is healthy, comfortable, well-nourished, safe, able to express innate behaviour, and if it is not suffering from unpleasant states such as pain, fear, and distress” [62]. The tendencies reported in Figure 4 reflect this definition, demonstrating the gradual convergence of the scientific community on topics that are perceived as crucial from relevant stakeholders. The documents analysed used both welfare and wellbeing to define the cow’s condition. However, the term “wellbeing” may recall only the positive side, while welfare is the most appropriate definition for the same concepts [63] Therefore, in this review, we have gathered both terms under the topic “welfare”. According to Figure 4, there has been a trend of increasing interest in animal health, but not as large as for animal behaviour. In general, the greater interest towards animal welfare, health and behaviour have shown an increasing tendency over time starting after 2004–2007, and this could be linked to the largest project on animal welfare, the FP6 Welfare Quality project that was going on during those years and gave a start to numerous publications in the following years in different production systems. The project has also shifted attention from resource and management-based measures to animal-based measures when assessing animal welfare [64]. Thus, more emphasis was given to the link between health and behaviour and how animal expression and behaviours can reveal precious information on their health and mental state [65,66]. It could also be discussed that the increased interest for animals (welfare, health and behaviour) followed the outset of antibiotic resistance and the pressure on farmers and veterinarians to use fewer pharmaceutical treatments. The aim was to implement more preventive measures and to have prompt reactions before needing to use antibiotics, and this was transferred to need for technology to help in quick diagnosis. However, as shown in Table 3, the research on animal welfare is still underdeveloped compared to the productive aspects.

The analysis of the tendencies of cluster “Process” considered both the milking process and the sustainability of milk production (Figure 5a,b) Although the milking time followed the same course of milk production over the years, its weight was lower. Most of the research papers were related to the objective of productive efficiency, which maximisation can be obtained by improving the performance of AMS. As the efficiency of AMS can be maximised by increasing the ratio milk flow/unit of time [67], the similar course of milk production and milking time was unequivocal. The interconnection between milk production and milking time was also disclosed by the results of the co-correlations analysis (Figure 6). The milking frequency, defined as the number of milking events per cow in any 24 h, is relatively flexible in AMS as the milking sessions are not strictly defined. Therefore, the milking frequency can be considered another performance indicator in AMS [50]. Figure 5a shows that, compared to milking time, the focus of research on milking frequency was lower but relatively stable over the observed period. By the matter of fact, it is straight forward that milking frequency is strongly related to both management and animal behaviour, but this is not reflected in Figure 6. It seems, therefore, that these subjects have not been exploited together, so far. This finding underlines a gap, and research needs to be carried out, along with the need for multidisciplinary approaches between scientists from the technology and animal sciences fields.

Along with the quantitative aspects, the quality was found to be critical. However, as the influence of AMS and increased milking frequency on milk quality is controversial [68,69,70], this topic would benefit from more consideration from the scientific community. The results of the tendencies of subjects concerning the sustainability of the process showed a growing interest in themes as the working environment and the natural environment (Figure 5b). According to a survey conducted by Salfer et al. [71], several farmers decided to adopt AMS to reduce long-term effects on milkers’ health. However, a negative perception is commonly associated with AMS as they imply the reduction of farms practising grazing. In the last years, various studies investigated the influence of AMS on environmental parameters to assess whether robot-assisted farms were suitable for sustainable production [72,73,74]. Our research highlighted that the interest in these topics is likely to keep growing in the coming years.

Concurrently with the focus on the animal and process aspects, the scientific community carried out continuous experimentation on the technologies and components related to AMS. Although these subjects showed lower frequency, their constant trend over the years proves the propensity of the research world to fulfil pioneering areas. The analysis of cluster “Technology” disclosed the gradual shift from data to DSS (Figure 5c). Detailed and local data collected by single farmers began to be used to derive general information for systemic control of processes. The use of herd DSS is spreading to fulfil the farmers’ need for automation and management of an increasingly complex sector [75]. Moreover, DSS represent a crucial control tool in precision livestock farming, thus supporting efficient and sustainable production. 

This review identified imaging and video technologies as critical sensing tools developed over the last years, with a sharp expansion starting from the 2008–2011 four-year period (Figure 5d). For example, vision systems have been used for teat detection and positioning [76], health monitoring [77], body condition [78] and animal behaviour surveys [79]. Imaging and video techniques contributed increasing the efficiency and precision of several production stages and are likely to be further implemented in the future. Sensing tools, data analytics and DSS generate significant amounts of interoperable data from Internet-connected farms. Combining full information may help in developing smarter managing solutions. Therefore, cloud-based farm analytics platforms will be a necessary medium for precision dairy farms. This tendency was disclosed in Figure 5d, where the recent interest of literature on the Internet of Things (IoT) is shown.

As regards the components, the high-frequency words displayed in Table 3 did not permit us to identify specific tendencies, despite the dominating relevance of somatic cell count as an indicator of milk quality.

The last analysis performed for the scientific literature was the detection of the interconnections of topics. As shown in Figure 6, the strongest co-correlations involved heart rate. Although only four papers included “heart rate” in their title, this parameter was used in several abstracts and used as keyword by several authors. Cardiovascular parameters are used as indicators of health and welfare of animals. Heart rate and its variability have been commonly used to assess stress status in dairy cows milked in AMS [80]. On the one hand, the high number of documents associating health and heart rate do not provide specific information on the research tendencies. On the other hand, the strong correlation between heart rate and concepts as “analysis”, “model” and “data” confirm the previous findings on the growing interest on animal welfare and new tools for its monitoring. Therefore, several studies related to the productive aspects used heart rate as cows’ health indicator [81]. As it can be observed in Figure 6, besides heart rate, some other words were found to be involved in multiple connections (e.g., time, milk production and cell count). However, most of these words are usually commonly used as keywords in papers on AMS. Therefore, their co-occurrence with several topics does not provide further information. 

As regards to the patents tendencies analysis, Figure 7 shows that the maximum number of patents was registered in 1998–1999 when the first Voluntary Milking System was commercialised, and Japan accessed the AMS research [82]. After a sharp decrease, the patents registration was stabilised, despite natural oscillations. When comparing this course with the papers course (Figure 1), the time lag between the industrial and academic research is clear. On the one hand, the industrial research’s maximum effort was spent in the initial time to allow the implementation of AMS suited to be adopted on a commercial scale. After the spread of AMS on a larger scale, academic research’s interest reached a peak and then stabilised. It may be concluded that the AMS sector represents one of the cases indicated by Meyer [83] in which the technological development preceded its scientific rationalization. The main contributors for patents where countries historically linked to AMS (the Netherlands and Sweden). Moreover, Figure 8 identified China as an emerging country for AMS patents.

As reported in Figure 9, the course of the four clusters varied over the last two decades. Initially, the focus of industrial research was on the technological implementation of the AMS. For example, new sensors were developed to support high-technology AMS, such as mastitis and abnormal milk detectors [25]. More recently, the focus shifted towards the improvement of the process efficiency. Over the last ten years, the target of patented research became the components. Table 4 describes a completely different approach to the AMS aspects of the patents compared to papers, with components and technology centralising the researchers’ attention.

Within the cluster “Components” (Figure 10a), the findings suggest that the industrial research invested the first decade working on teat cups. The second decade shifted the focus on storage systems and capacity, as suggested from the increasing interest on pumps and tanks. The implementation of the milking arm was another trending topic. This tendency may be related to the use of the hydraulic arms, which allow increasing productivity and efficiency. Based on this new technology, recent developments implemented silent and efficient milking arms reduce the disturbance to cows. Thanks to the active patented research, the milking arm is characterised by high flexibility and precision for detecting teats.

The recent growing focus on arm motion was confirmed by the course analysis of cluster “Technology” (Figure 10b). As for papers, imaging techniques were largely adopted by the industry, and the tendency is likely to grow in the future. Although models were not included within the highest frequency word shown in Table 4, their increase starting from 2008–2011 was remarkable. This finding confirms that both scientific and patented research seeks to fulfil the farmers’ need for management DSS.

Despite some fluctuation, the analysis of cluster “Process” stressed the relevance of patents covering the management of cleaning operations (Figure 10c). Milking hygiene is crucial for obtaining high-quality milk and preventing the appearance of mastitis [45]. According to Hogenboom et al. [84], adequate teat sanitation is not always ensured in AMS, and teat cleaning failures are usual. Our findings suggest that industrial research is trying to perfect the cleaning systems, but the upward trend of the related topics may indicate that more research should be expected in this sector. According to Figure 10c, the effort on vacuum-technologies raised over the last twenty years. Research on pulsation technologies contributed to the improvement of barns efficiency. Indeed, the pulsation ratio influences milk flow rate, milking time and udder health [85].

Cluster “Animal” showed the lowest frequency within the patent’s tendencies. Figure 10d shows that initially patents were focused on the performing of the feeding systems, designed to attract cows for voluntary milking. More recently, the focus became the implementation of barn environment. Simultaneously, the industry responded to the need for maintaining high-quality production and ensuring animal welfare through the perfection of health-monitoring equipment, such as automated weighing scales [86] and optical-based techniques [87].

The quantitative review of papers and tendencies highlighted that the interests of the scientific and industrial research are complementary. On the one hand, the scientific community focused the main activity on animal welfare and productivity. Moreover, the improvement of the efficiency and sustainability of the process was constantly pursued. On the other hand, the primary objectives of the industrial research were to implement adequate components and technologies to sustain a steadily growing sector. Nevertheless, the purposes of the research were similar, aiming at achieving sustainable production, in compliance with the needs of animals and taking advantage of new sensing techniques. 

## 5. Conclusions

The quantitative review proposed in this study addressed for the first time a global assessment of the state of the art of research on AMS. The scientific and industrial approach to AMS was analysed through the examination of patents and papers of the last 20-years. This analysis enabled to identify tendencies and gaps of research in the AMS sector.

The high number of documents examined in this study allowed the identification of the tendencies and gaps of research, thus allowing future targeted development. Quantitative reviews allow a global perspective of scientific and industrial research. Nevertheless, it should be considered that the analysis of single words implies the interpretation of the topics analysed by the authors. To address this issue, our findings were supported by in-depth analysis of single studies, which were reported to help to interpret the quantitative results.

Significant advances in sensing techniques and process management were assessed. Moreover, the implementation of herd DSS based on farm data availability indicates the competitiveness of the AMS sector. 

Despite the recent growing trends of topics related to animal welfare and environmental sustainability, further progress will be needed to meet the stakeholders’ demand for ethically correct production. Similar considerations may be considered for the process priorities, which should focus more on milk quality.

The findings of this review should stimulate scientific research towards deficient topics, thus contributing to the improvement of the AMS.

## Figures and Tables

**Table 1:** The custom queries used for documents extraction (ar = article; re = review; ch = book chapter).

Document Type	Search Query	Database
Papers	(TITLE-ABS-KEY(“automatic milking”) OR TITLE-ABS-KEY(“milking robot”) OR TITLE-ABS-KEY(“robotic milking”) OR TITLE-ABS-KEY(“automated milking”) OR TITLE-ABS-KEY(“automatically milking”)) AND (LIMIT-TO(DOCTYPE, “ar”) OR LIMIT-TO(DOCTYPE, “re”) OR LIMIT-TO(DOCTYPE, “ch”)) AND (LIMIT-TO(PUBYEAR, 2019) OR LIMIT-TO(PUBYEAR, 2018) OR LIMIT-TO(PUBYEAR, 2017) OR LIMIT-TO(PUBYEAR, 2016) OR LIMIT-TO(PUBYEAR, 2015) OR LIMIT-TO(PUBYEAR, 2014) OR LIMIT-TO(PUBYEAR, 2013) OR LIMIT-TO(PUBYEAR, 2012) OR LIMIT-TO(PUBYEAR, 2011) OR LIMIT-TO(PUBYEAR, 2010) OR LIMIT-TO(PUBYEAR, 2009) OR LIMIT-TO(PUBYEAR, 2008) OR LIMIT-TO(PUBYEAR, 2007) OR LIMIT-TO(PUBYEAR, 2006) OR LIMIT-TO(PUBYEAR, 2005) OR LIMIT-TO(PUBYEAR, 2004) OR LIMIT-TO(PUBYEAR, 2003) OR LIMIT-TO(PUBYEAR, 2002) OR LIMIT-TO(PUBYEAR, 2001) OR LIMIT-TO(PUBYEAR, 2000)) AND (LIMIT-TO(LANGUAGE, “English”))	Scopus
Patents	ti = “Automatic milking” OR ti = “Milking robot” OR ti = “robotic milking” OR ti = “automated milking” OR ti = “automatically milking”	EspaceNet

**Table 2:** Overview of the reviews yielded from 2000 to 2019 in the automatic milking systems (AMS) sector.

Title	Topic	Summary	Tipology	Year	Reference
Food biotechnologies and retail ethics: A survey of UK retailers’ views on the use of two dairy technologies	Technology	Retailers’ perception of AMS acceptability	questionnaire	2001	[34]
Automatic on-line analysis of milk constituents (urea, ketones, enzymes and hormones) using biosensors	Technology	Monitoring of animal health and milk quality parameters in automated milking farms using biosensors	qualitative	2002	[35]
Sensors and management support in high-technology milking	Technology	Sensors for the detection of abnormal milk and mastitis in high-tech farms	qualitative	2003	[25]
Indicators of inflammation in the diagnosis of mastitis	Animal	New mastitis detection systems in automated milking farms	qualitative	2003	[36]
Main issues in robotic milking of cows	Process/Animal	Quantification of the AMS in terms of production, quality and animal health	meta-analysis	2006	[37]
Impacts of automatic milking systems on milk cooling and their according technical solutions	Process	Different AMS cooling approaches	qualitative	2006	[38]
Automatic milking: State of the art: Current and future developments	Process	Overview of the development of AMS	qualitative	2006	[39]
External and internal damage of cow teats	Animal/Technology	Methodologies and technologies for teat. Comparison of teat conditions in AMS and conventional milking systems	qualitative	2007	[40]
Induction of milk ejection and milk removal in different production systems	Animal	Analysis of the milk ejection process with a comparison between AMS and conventional milking systems	qualitative	2008	[41]
Pros and cons of automatic milking in Europe	Process/Animal	Focus on the pros and cons of AMS as regards milking frequency, quality, cow traffic, and animal welfare	qualitative	2008	[21]
Systems in organic dairy production	Process	Investigation of the stakeholders’ perception of the contribution of AMS to sustainability in organic dairy production	questionnaire	2008	[42]
Sensors and clinical mastitis-the quest for the perfect alert	Animal/Technology	Analysis of several sensor-based models for clinical mastitis detection	qualitative	2010	[43]
The mathematical description of lactation curves in dairy cattle	Technology	Overview of functions for modelling of lactation curves	qualitative	2011	[44]
Invited review: Udder health of dairy cows in automatic milking	Animal	Focus on udder health and cow and milking management in automated milking farms	qualitative	2011	[45]
Invited review: Effect of udder health management practices on herd somatic cell count	Animal	Analysis of the relationships between management practices and herd somatic cell count	systematic	2011	[46]
Mastitis control in robotic milking systems	Animal	Focus on the pros and cons of AMS as regards mastitis and milk quality	qualitative	2012	[47]
Invited review: The impact of automatic milking systems on dairy cow management, behavior, health, and welfare	Animal	Analysis of the effects of AMS on milk quality and animal health and welfare	qualitative	2012	[10]
Effect of lameness on the behavior of dairy cows under intensive production systems	Animal	Investigation on the impact of lameness on the behavior of dairy cows	qualitative	2012	[48]
Comparative analysis on effectiveness of AMS use on an example of three European countries	Process	Comparison of technical, biological, economic and technological data of European automated milking farms	quantitative	2013	[49]
Milking frequency management in pasture-based automatic milking systems: A review	Process	Comparison of different factors influencing milking frequency and interval in pasture-based AMS	qualitative	2014	[50]
Grazing increases the unsaturated fatty acid concentration of milk from grass-fed cows: A review of the contributing factors, challenges and future perspectives	Process	Investigations of the effects of grazing in fatty acid composition of milk	qualitative	2015	[51]
Factors determining the susceptibility of cows to mastitis and losses incurred by producers due to the disease—A review	Animal	Overview of the factors influencing the susceptibility of cows to mastitis	qualitative	2015	[52]
Systemic perspectives on scaling agricultural innovations. A review	Technology/Process	Connections between technologies, processes and practices within innovative agricultural models	systematic	2016	[53]
Early detection of clinical mastitis from electrical conductivity data in an automatic milking system	Animal	Overview of the indexes and algorithms for the early detection of clinical mastitis	qualitative	2017	[54]
Innovation, practical benefits and prospects for the future development of automatic milking systems	Process	Overview of the development of AMS	qualitative	2017	[24]
Automatic milking systems-factors involved in growing popularity and conditions of effective operation literature review	Process/Animal	Investigation of the benefits of AMS from the human and animal perspective	qualitative	2018	[55]
Mastitis Control in Automatic Milking Systems	Animal/Technology	Review of the technologies and methodologies for mastitis detection	qualitative	2018	[56]
Designing Automated Milking Dairy Facilities to Maximize Labor Efficiency	Process	Analysis of the influence of barn design on the labor efficiency	qualitative	2019	[57]

**Table 3:** Conceptual clusters derived from the analysis of papers. The main words of each cluster, along with their frequency, is reported in the second column. Words marked with an asterisk have undergone the stemming process, which consists of including in a single lemma all variant forms of the same word (i.e., images and imaging, or udder and teats). In most cases stemming was applied to words with common root (e.g., lactation and lactating); in some cases, stemming was applied to words with the same meaning (e.g., udder and teat).

Cluster	Main Words and Relative Occurrence			Cluster Weight
Animal	Cattle/Herd (9.1%)	Lactation * (6.4%)	Behaviour (4.1%)	49%
	Udder * (7.8%)	Mastitis (4.5%)	Barn/Stall * (4.1%)	
	Feed * (7.6%)	Forage * (4.4%)	Welfare (3.2%)	
Process	Milk Prod. * (18.5%)	Management * (5.5%)	Milk Quality (3.6%)	27%
	Time (12.6%)	Milk Flow (4.8%)	Air Pollution * (3.3%)	
	Measurement (5.6%)	Milk Frequency (4.7%)	Milk. Interval (3.1%)	
Technology	Data (12.7%)	Detection * (8.8%)	Development * (4.4%)	17%
	Analysis * (11.4%)	Recording * (6.9%)	Cow Traffic (3.9%)	
	Model * (10.6%)	Sensors * (5.8%)	Monitoring * (3.9%)	
Components	Cell count * (40.6%)	Tandem (1.5%)	Tube (1.0%)	7%
	Volount. MS (26.6%)	Reservoir (1.0%)	Paddock (1.0%)	
	Parlour (14.0%)	Rotary (1.0%)	Alarms (0.9%)	

**Table 4:** Conceptual clusters derived from the analysis of patents. The main words of each cluster, along with their frequency, is reported in the second column. Words marked with an asterisk have undergone the stemming process, which consists of including in a single lemma all variant forms of the same word (i.e., images and imaging, or udder and teats). In most cases stemming was applied to words with common root (e.g., analysis and analyse); in some cases, stemming was applied to words with the same meaning (e.g., udder and teat).

Cluster	Main Words and Relative Occurrence			Cluster Weight
Components	Teat cups (29.0%)	Tank (7.1%)	Parlour (4.4%)	30%
	Arm (14.0%)	Valve (5.6%)	Pump (3.6%)	
	Pipe/Pipeline (7.7%)	Rotary (4.7%)	Storage (3.3%)	
Technology	Device (33.2%)	Receiver * (5.2%)	Sensor * (3.6%)	29%
	Connection (17.8%)	Electromagnetic (4.1%)	Analysis * (3.2%)	
	Imaging * (12.3%)	Computer (3.9%)	Signal (2.6%)	
Process	Control * (19.4%)	Water (6.3%)	Pulsation * (4.8%)	25%
	Vacuum (10.0%)	Cooling * (6.0%)	Detection * (3.8%)	
	Measurement * (7.4%)	Clean/Wash * (5.0%)	Air/Air Intake (3.7%)	
Animal	Udder * (42.2%)	Body/Weight (9.1%)	Health * (2.2%)	16%
	Barn/Stall * (21.7%)	Cattle/Herd (5.5%)	Chemical * (1.6%)	
	Feeding * (11.4%)	Quarter (4.1%)	Colostrum (1.6%)	

## Data Availability

The data presented in this study are available on request from the corresponding author.
